# Gut microbiota disturbance disrupts osteonal organization and mechanical competence during postnatal cortical bone maturation in rabbits with local metabolic and transcriptional remodeling

**DOI:** 10.3389/fcell.2026.1937899

**Published:** 2026-10-05

**Authors:** Xiaopei Yang, Ziming Geng, Hongji Wang, Gongzi Zhang, Shicheng Luo, Binbin Liu, Yansen Wang, Shuwei Zhang, Lihai Zhang

**Affiliations:** 1 Department of Orthopedics, Chinese PLA General Hospital, Beijing, China; 2 Department of Rehabilitation Medicine, Chinese PLA General Hospital, Beijing, China; 3 Health Sciences Institute, China Medical University, Shenyang, China

**Keywords:** antibiotic-induced microbiota disturbance, bone quality, cortical bone, gut microbiota, metabolomics, osteon, rabbit model, transcriptomics

## Abstract

**Introduction:**

Bone strength depends not only on bone mass and mineral density but also on cortical tissue organization and material quality. Osteons are key structural units of cortical bone, yet whether gut microbiota disturbance affects cortical bone maturation at the osteon level remains unclear.

**Methods:**

We first characterized cortical canal remodeling and osteonal organization in New Zealand rabbits at 1, 2, and 3 months of age. Antibiotic-induced microbiota disturbance (AIMD) was then applied during this early osteon-forming window, followed by assessments of gut microbiota composition, osteon morphology, cortical bone micro-CT parameters, mechanical performance, femoral metabolomics, and cortical bone transcriptomics.

**Results:**

AIMD reduced gut microbial diversity and altered microbial community structure. At 3 months of age, AIMD-treated rabbits showed impaired osteonal organization, including a reduced proportion of secondary osteons and altered osteon morphology. These osteon-level changes were accompanied by decreased maximum load and stiffness, whereas conventional micro-CT parameters of cortical geometry and mineral density remained unchanged. Multi-omics analyses further revealed local metabolic and transcriptional remodeling involving lipid-related metabolites, bone matrix regulation, osteocyte communication, and metabolic enzyme programs.

**Discussion:**

These findings suggest that early gut microbiota disturbance impairs osteon-level cortical bone maturation and reduces mechanical competence. Osteonal organization may therefore represent a sensitive layer of cortical bone quality affected by gut microbiota disturbance during postnatal skeletal development.

## Introduction

Bone strength reflects not only bone mass and bone mineral density (BMD), but also tissue organization and material quality (Morgan et al., 2018). Although BMD and trabecular microarchitecture are widely used to evaluate skeletal health, cortical bone is a major determinant of long-bone mechanical competence (Morgan et al., 2018; Isojima and Sims, 2021). Within the cortex, osteons and intracortical canal systems form key structural units involved in tissue remodeling, matrix organization, vascular integration and load bearing (Chang and Liu, 2022). Osteon-level organization therefore represents an important, but often underappreciated, dimension of cortical bone quality.

Osteonal organization is progressively established during postnatal cortical bone maturation. During this process, intracortical vascular canals are incorporated into primary osteons, whereas secondary osteonal structures emerge with ongoing remodeling (Chang and Liu, 2022; Pazzaglia et al., 2010; Pazzaglia et al., 2007). Rabbits provide a useful model for studying this process because their long bones develop prominent intracortical canals and osteonal systems (Pazzaglia et al., 2010; Pazzaglia et al., 2007). Unlike mice, in which physiological intracortical remodeling is limited and prominent secondary osteonal systems are generally absent, rabbits develop well-defined intracortical vascular canals and secondary osteons during postnatal growth (Pazzaglia et al., 2010; Pazzaglia et al., 2007; Koh et al., 2024). This feature provides a useful model for assessing osteonal changes associated with antibiotic-induced microbiota perturbation during cortical tissue maturation, which are difficult to resolve in conventional mouse models. Postnatal maturation of rabbit cortical bone is also accompanied by progressive changes in mineral deposition, collagen organization and tissue mechanical properties (Isaksson et al., 2010). More recent four-dimensional imaging studies have further demonstrated active longitudinal progression of basic multicellular units within rabbit cortical bone (Harrison et al., 2022). However, the early developmental window during which osteonal organization is established, and the susceptibility of this process to systemic environmental perturbations, remain incompletely defined.

The gut microbiota has emerged as an important regulator of skeletal development and bone homeostasis through immune, endocrine and metabolic pathways (Lyu et al., 2023; Liu et al., 2026). Germ-free animal experiments have demonstrated that the presence or absence of the gut microbiota can substantially influence bone mass and osteoclast-related immune activity (Sjögren et al., 2012). The gut microbiota and its metabolites can also stimulate systemic insulin-like growth factor 1 signaling and promote bone formation and skeletal growth (Yan et al., 2016). Experimental antibiotic-induced perturbation of the gut microbiota has been shown to disrupt osteoimmune communication and alter bone development and remodeling (Hathaway-Schrader et al., 2019). The direction and magnitude of these skeletal effects may nevertheless depend on developmental stage, sex, treatment duration and the specific antibiotic regimen employed (Lyu et al., 2023; Liu et al., 2026; Hathaway-Schrader et al., 2019). Most studies of the gut microbiota–bone axis, however, have focused on BMD, trabecular microarchitecture, serum bone-turnover markers or general indices of remodeling (Lyu et al., 2023; Liu et al., 2026; Sjögren et al., 2012; Yan et al., 2016; Hathaway-Schrader et al., 2019).

When cortical bone has been examined, it has generally been assessed using gross micro-CT parameters, such as cortical thickness, area and mineral density. In several microbiota-related intervention models, trabecular bone changes were detected without corresponding alterations in these cortical indices (Hathaway-Schrader et al., 2019; Carson et al., 2023; Rios-Arce et al., 2020). However, preserved cortical geometry and mineral density do not establish that cortical maturation is unaffected, because these measures do not directly capture the organization of intracortical canals and osteons. Whether gut microbiota disturbance disrupts osteonal organization during cortical bone maturation therefore remains unknown.

In this study, we first characterized early postnatal cortical bone development in New Zealand rabbits to identify a window of intracortical canal remodeling and osteonal organization. We then induced gut microbiota disturbance with antibiotics during this window and assessed gut microbiota composition, osteon morphology, cortical bone micro-CT parameters, mechanical performance, femoral cortical bone metabolomics and cortical bone transcriptomics. We aimed to determine whether early gut microbiota disturbance disrupts osteonal organization during cortical maturation and to characterize the local metabolic and transcriptional changes associated with this process.

## Materials and methods

### Animals and sample collection

Male New Zealand white rabbits were used to define the early postnatal cortical bone developmental window and to evaluate the effect of antibiotic-induced microbiota disturbance (AIMD) on early osteon formation. Animals were housed under controlled conditions at TongHeLitai Biotechnology Co., Ltd (China, Beijing). All animal procedures were approved by the ethics committee of TongHeLitai Biotechnology Co., Ltd.

For developmental-window analysis, femoral cortical bone samples were collected from rabbits at 1, 2 and 3 months of age. These samples were used for histological observation and quantitative assessment of vascular canal density. Osteon area was quantified only in 2- and 3-month-old samples with identifiable osteonal structures according to the predefined morphological criteria.

For the AIMD experiment, rabbits were assigned to Control or AIMD groups beginning at 1 month of age and maintained until sample collection at 3 months of age. At the endpoint, fresh fecal samples were collected for 16S rRNA gene sequencing. Femurs were harvested for cortical bone histology, micro-CT, three-point bending, metabolomics, RNA sequencing (RNA-seq) and reverse transcription quantitative polymerase chain reaction (RT-qPCR) analyses. Osteon morphometry and mechanical testing were performed using five animals per group, whereas matched microbiome, metabolomics and transcriptomics analyses were performed using a subset of three animals per group with complete paired multi-omics data.

Rabbits were euthanized by an overdose of pentobarbital sodium via the marginal ear vein. Then, femurs were dissected free of surrounding soft tissue. Femoral samples used for histology, micro-CT and three-point bending tests were preserved in paraformaldehyde (PFA) solution until downstream processing and measurement. Cortical bone samples for metabolomics and RNA extraction were rapidly isolated, snap-frozen in liquid nitrogen and stored at −80 °C until analysis.

### Antibiotic-induced microbiota disturbance

AIMD was induced from 1 to 3 months of age to disturb the gut microbiota during the early osteon-forming window. Rabbits in the AIMD group received an antibiotic cocktail containing rifaximin, neomycin and metronidazole (MB1492, MB1716 and MB2200, respectively; Meilunbio, Dalian, China) at doses of 80 mg/kg, 100 mg/kg and 30 mg/kg, respectively, via drinking water. This regimen was selected to provide broad-spectrum intestinal antimicrobial coverage and induce broad gut microbiota disturbance rather than to target a single bacterial taxon. It was adapted from antibiotic-based microbiota depletion strategies commonly used in animal models, in which neomycin and metronidazole are frequently included as components of broad-spectrum antibiotic cocktails (Tan et al., 2022; Zarrinpar et al., 2018). Rifaximin was included as a poorly absorbed intestinal antibiotic known to alter gut microbial composition (Poteres et al., 2020). The antibiotic-containing drinking water was freshly prepared and replaced every day. Control rabbits received untreated drinking water over the same period. Body condition, general health status and overt behavioral changes, including persistent lethargy or inactivity, were monitored throughout the intervention. No obvious behavioral abnormalities were observed in either group during routine monitoring.

### Hard-tissue resin embedding and sectioning

Femoral bone samples were fixed in 4% paraformaldehyde (G1101; Servicebio, Wuhan, China) for at least 48 h. After fixation, samples were dehydrated through a graded ethanol series, followed by absolute ethanol and xylene treatment. The dehydrated specimens were infiltrated with methyl methacrylate-based resin (M813511; Macklin) solutions at 4 °C and then embedded in resin under vacuum. Polymerization was performed at 37 °C. Undecalcified hard-tissue sections were cut at 10 μm using a hard-tissue microtome equipped with a tungsten carbide blade and dried overnight at 60 °C before staining.

### Toluidine blue staining and histological imaging

Resin sections were deplasticized and rehydrated before staining. Briefly, sections were treated with ethylene glycol monoethyl ether acetate, passed through graded ethanol solutions and rinsed in water. Sections were stained with toluidine blue (G1032; Servicebio, Wuhan, China) for 5 min, rinsed, and differentiated with 1% acetic acid when necessary. After rapid dehydration and clearing in xylene, sections were mounted with neutral resin. Histological images were acquired using a bright-field microscope, and cortical bone regions were used for subsequent osteon morphology analysis (Peev et al., 2024).

### Cortical ROI definition and osteon morphometry

Dorsal, ventral, medial and lateral cortical regions of interest (ROIs) were predefined on each femoral transverse section (Pazzaglia et al., 2010). Vascular canal density was calculated as the number of intracortical vascular canals per ROI area and expressed as n/mm^2^. For developmental-window analysis, vascular canal density was quantified from 1- to 3-month-old samples, whereas osteon area was quantified only in 2- and 3-month-old samples with identifiable osteonal structures.

Primary osteons were defined as vascular canal-centered structures surrounded by organized concentric matrix deposition but lacking a distinct peripheral cement line. Secondary osteons were identified as intracortical structures consisting of a central Haversian canal, surrounding concentric lamellae and a recognizable peripheral cement line, according to established histomorphological criteria. Secondary osteon percentage was calculated as the number of secondary osteons divided by the total number of identifiable osteons (Chang and Liu, 2022; Pazzaglia et al., 2007). Osteon area, roundness and other morphometric parameters were measured using ImageJ (20). For each animal, measurements were first averaged within each ROI and then summarized at the animal level for statistical analysis.

### Micro-CT analysis

Femoral samples were removed from fixative, gently blotted to remove excess liquid and scanned using a NMC-200 Micro-CT system (PINGSENG Healthcare Technology, China) in continuous scanning mode. Scanning was performed at 80 kV and 0.06 mA, with a frame rate of 20 frames/s and a total of 4,000 projection frames. The source-to-detector and source-to-object distances were 410 and 90 mm, respectively. The transverse and axial fields of view were 51 and 16.014 mm, respectively, and images were reconstructed with an isotropic voxel size of 51 μm using a Feldkamp–Davis–Kress (FDK) reconstruction algorithm in Recon software. For cortical bone analysis, a 5-mm-long region centered at the midpoint of the femoral shaft was defined as the region of interest (ROI) and applied consistently to all samples. Mineralized cortical bone was segmented in Avatar software using a fixed CT threshold of 1106. Cortical thickness, cortical area and tissue mineral density were subsequently quantified and exported for statistical analysis.

### Three-point bending test

Cortical bone mechanical performance was assessed using a three-point bending test. Mid-diaphyseal cortical bone specimens were prepared from the same anatomical region of each femur. Samples were cut at low temperature and low speed using an EXAKT diamond saw to minimize thermal and mechanical damage during preparation. Each specimen was divided into four beam-like cortical bone strips of approximately 3 mm × 18 mm. Each beam was placed individually on the three-point bending fixture and loaded until fracture using a Shimadzu AGX-S testing system (Shimadzu, Japan). The support span was set to 14 mm, and the loading rate was 2 mm/min. Load-displacement data were recorded automatically by the testing system. Maximum load and stiffness were calculated from the recorded force-displacement curves. The four beam specimens from each animal were averaged to obtain one animal-level value for statistical analysis.

### 16S rRNA gene sequencing and microbiota analysis

Fecal microbial DNA was extracted using the Stool and Intestinal Content DNA Extraction Kit (DP328; TIANGEN Biotech, Beijing, China) according to the manufacturer’s instructions. The V3-V4 region of the bacterial 16S rRNA gene was amplified using primers 341F (CCTAYGGGRBGCASCAG) and 806R (GGACTACNNGGGTATCTAAT). PCR amplification was performed in a 30 µL reaction containing 15 µL Phusion High-Fidelity PCR Master Mix, 1 µL forward primer (0.2 µM), 1 µL reverse primer (0.2 µM), 10 µL genomic DNA (1 ng/μL, 10 ng total) and nuclease-free water. The PCR program consisted of initial denaturation at 98 °C for 1 min, followed by 30 cycles of 98 °C for 10 s, 50 °C for 30 s and 72 °C for 30 s, with a final extension at 72 °C for 5 min.

Amplicon libraries were prepared using the NEBNext Ultra II DNA Library Prep Kit (E7645B; New England Biolabs, Beijing, China) and sequenced on an Illumina NovaSeq 6000 platform (Illumina, San Diego, CA, United States). Raw sequencing data were processed using a DADA2-centered QIIME2 workflow (Bolyen et al., 2019; Callahan et al., 2016). Briefly, demultiplexed reads were quality filtered, denoised, paired-end merged and chimera-removed to generate amplicon sequence variants (ASVs). Taxonomic classification was performed using a naive Bayes classifier trained against the SILVA database (Bokulich et al., 2018; Quast et al., 2013). Endpoint fecal samples from Control (C1-C3) and AIMD (I1-I3) rabbits were used for downstream microbiota analysis. Samples were rarefied to an even sequencing depth of 37,884 reads per sample before diversity analyses. Alpha diversity was evaluated using Shannon index and richness indices, including observed ASVs and Chao1. Welch’s t-test was used as the primary statistical test. Beta diversity was assessed using Bray-Curtis dissimilarity and visualized by principal coordinate analysis (PCoA). Group effects were evaluated using permutational multivariate analysis of variance (PERMANOVA), and homogeneity of dispersion was assessed using betadisper. Genus-level community composition, linear discriminant analysis effect size (LEfSe) analysis with linear discriminant analysis (LDA) scores, and z-score heatmaps of representative differential taxa were used to characterize AIMD-associated microbial shifts. (Segata et al., 2011).

### Untargeted metabolomics of femoral tissue

Femoral cortical tissue samples from the AIMD and Control groups were subjected to untargeted liquid chromatography–tandem mass spectrometry (LC-MS/MS)-based metabolomics. Briefly, tissue samples were ground in liquid nitrogen, extracted with prechilled 80% methanol, incubated on ice, and centrifuged at 15,000 g at 4 °C. The supernatant was diluted with LC-MS-grade water to a final methanol concentration of 53%, centrifuged again, and injected into the LC-MS/MS system. Chromatographic separation was performed on a Vanquish ultra-high-performance liquid chromatography (UHPLC) system using a Hypersil Gold C18 column (100 × 2.1 mm, 1.9 μm) with a 12-min gradient at a flow rate of 0.2 mL/min. Metabolites were detected on an Orbitrap Exploris 120 mass spectrometer in both positive- and negative-ion modes.

Pooled quality-control samples were prepared by mixing equal aliquots of all experimental samples and were injected throughout the analytical run to monitor system stability. Positive- and negative-ion mode data were processed separately. Raw MS data were processed using XCMS for peak detection, retention-time correction, peak alignment and peak-area integration (Smith et al., 2006). Metabolite identification was performed by matching adduct ions, accurate mass information and MS/MS spectra against the Novogene in-house NovoMetDB database, with a mass tolerance of 10 ppm, followed by annotation using Kyoto Encyclopedia of Genes and Genomes (KEGG), Human Metabolome Database (HMDB) and LIPID MAPS (Kanehisa and Goto, 2000; Sud et al., 2007). Features with more than 50% missing values were removed, and remaining missing values were imputed using K-nearest neighbors. Background ions were removed according to blank samples. Relative metabolite abundance was represented by normalized peak area, and features with a coefficient of variation greater than 30% in quality-control (QC) samples were excluded.

For the study-specific analysis, only femoral cortical tissue samples were used, including three AIMD samples and three Control samples; serum metabolomics data and trabecular-bone columns were excluded. Positive- and negative-ion modes were processed separately, and annotated metabolites were integrated after annotation and sample matching for downstream biological interpretation. Principal component analysis (PCA) was performed using the normalized metabolite intensity matrix. Differential metabolite analysis was performed on log2 (intensity + 1) values using Welch’s t-test, and metabolites with P < 0.05 and |log2 fold change| ≥ 0.5 were selected as candidate differential metabolites for exploratory downstream analyses. Differential metabolites were visualized using volcano plots and z-score heatmaps, and metabolite class distributions were summarized based on HMDB, KEGG and LIPID MAPS annotations. Selected metabolites were further displayed using normalized peak areas.

### RNA-seq and differential expression analysis

Total RNA was extracted from femoral cortical bone using the TRNzol Universal Reagent (DP424; TIANGEN Biotech, Beijing, China) according to the manufacturer’s protocol. RNA integrity was assessed using an Agilent 5400 system (Agilent Technologies, United States), and RNA concentration was quantified using a Qubit 2.0 fluorometer (Invitrogen, United States). RNA-seq libraries were prepared using the Fast RNA-seq Lib Prep Kit V2 (RK20306; ABclonal Technology, Wuhan, China) and sequenced on an Illumina NovaSeq X Plus platform (Illumina, United States) to generate paired-end reads.

Raw sequencing reads were filtered to remove adaptor sequences, reads containing ambiguous bases and low-quality reads. Clean reads were aligned to the *Oryctolagus cuniculus* reference genome OryCun2.0 from Ensembl release 107 using HISAT2(30, 31). Gene-level read counts were obtained using featureCounts. Gene expression levels were summarized as read counts and fragments per kilobase of transcript per million mapped reads (FPKM) values. Principal component analysis was performed using the whole-transcriptome FPKM expression matrix. Differential expression between AIMD and Control cortical bone samples was analyzed from the gene count matrix using DESeq2 (32). For the final analyses in this study, genes with |log2 fold change| > 1 and P < 0.05 were retained as candidate differentially expressed genes (DEGs) for exploratory downstream analyses. DESeq2-adjusted P values for these candidate DEGs are provided in Supplementary Table S5. Genes without annotated gene symbols were excluded from curated downstream interpretation when necessary.

### Reverse transcription and quantitative polymerase chain reaction

Total RNA was extracted from cortical bone tissue. Approximately 20 mg of tissue was homogenized in RNA extraction reagent using zirconia grinding beads and a cryogenic tissue grinder. After phase separation, RNA was precipitated with isopropanol, washed with 75% ethanol and dissolved in RNA dissolution buffer. RNA concentration and purity were assessed using a NanoDrop 2000 spectrophotometer. Reverse transcription was performed using SweScript All-in-One RT SuperMix for qPCR with gDNA remover (G3335-50; Servicebio, Wuhan, China). The reverse-transcription program was 25 °C for 5 min, 42 °C for 30 min and 85 °C for 5 s.

qPCR was performed using 2× Universal Blue SYBR Green qPCR Master Mix on a Bio-Rad CFX Connect real-time PCR system. Each reaction contained SYBR Green master mix, forward and reverse primers, cDNA and nuclease-free water, and each sample was run in technical triplicate. The amplification program was 95 °C for 30 s, followed by 40 cycles of 95 °C for 15 s and 60 °C for 30 s. A melting-curve analysis was performed from 65 °C to 95 °C. Relative gene expression was calculated using the 2^−ΔΔCt^ method after normalization to the internal reference gene. Primer sequences for qPCR are available on request (Livak et al., 2001).

### Integrative analysis of transcriptome and metabolome

Integrative analysis was performed using the six matched cortical-bone samples with both RNA-seq and metabolomics data. The top 100 named DEGs were selected after excluding genes without annotated gene symbols, and all differential metabolites from the femoral cortical-bone metabolomics analysis were included. Gene expression and metabolite abundance were transformed as log2(FPKM + 1) and log2 (peak area + 1), respectively. Pearson correlation coefficients were calculated between DEGs and differential metabolites across the matched samples. KEGG compound, gene and pathway annotations were used to identify shared pathway contexts, and KEGG Markup Language (KGML) information was used when available to support gene-metabolite-pathway network construction.

### Statistical analysis

Each animal was considered an independent biological replicate. Data are presented as mean ± standard error of the mean (SEM) unless otherwise indicated. Welch’s t-test was used for two-group comparisons. For beta diversity, Bray-Curtis distances were analyzed by PERMANOVA, and betadisper was used to assess within-group dispersion. Pearson correlation was used for transcriptome-metabolome integration. Except for the exploratory omics feature-screening analyses described above, a two-sided P < 0.05 was considered statistically significant.

## Results

### Progressive osteonal organization during early postnatal cortical bone development in rabbits

To characterize early postnatal osteonal development during the period covered by the AIMD intervention, we performed histological observation and quantitative analysis of femoral cortical bone from 1-, 2- and 3-month-old New Zealand rabbits. To reduce bias from single-field selection, four cortical regions of interest (ROIs), including dorsal, ventral, medial and lateral regions, were predefined on femoral cross-sections and used for subsequent morphological assessment and quantitative analysis (Figure 1A).

**FIGURE 1:**
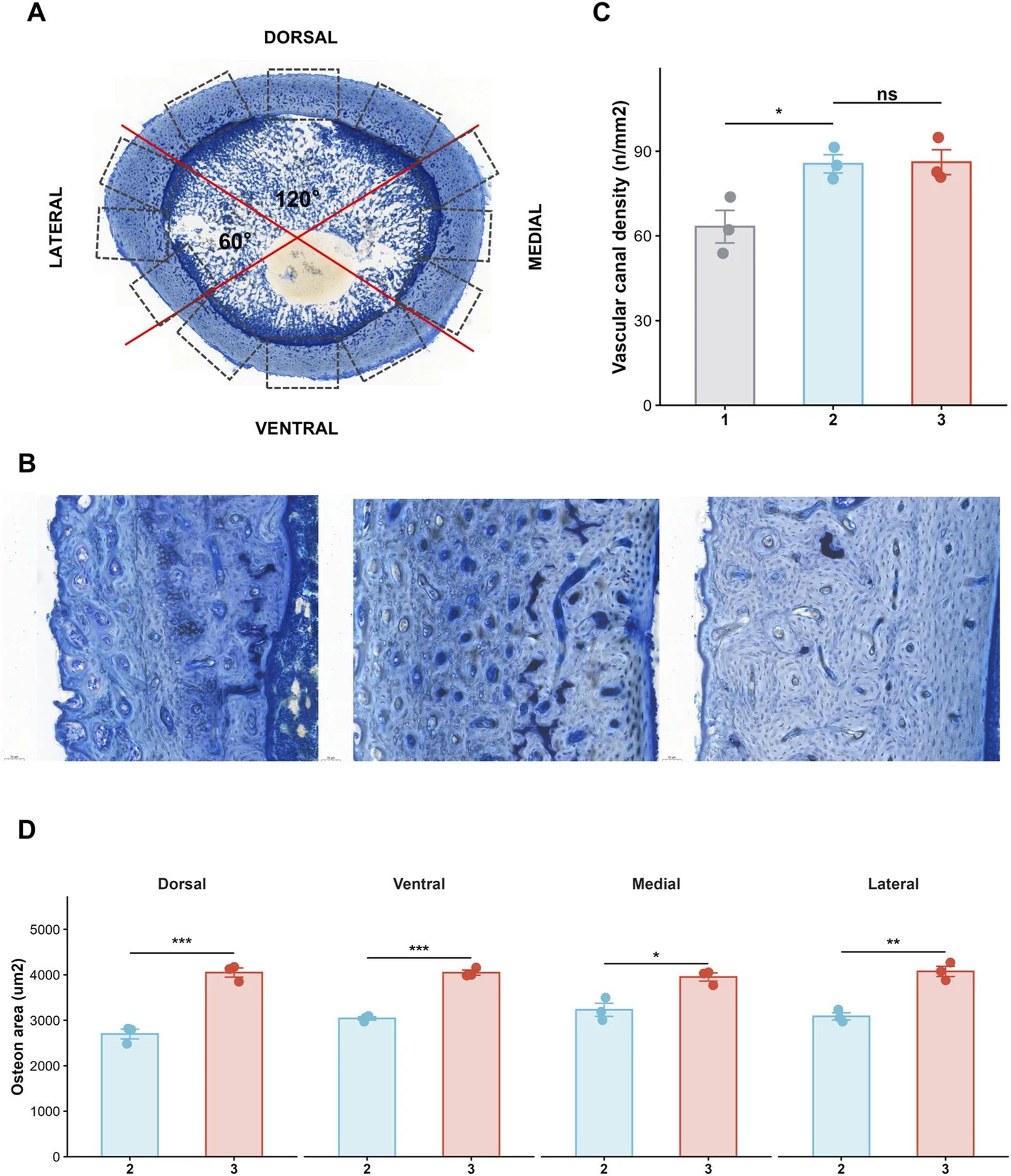
Early postnatal cortical canal remodeling and osteonal organization in rabbit femoral cortical bone. **(A)** Schematic illustration of the predefined cortical regions of interest (ROIs) used for histological analysis, including dorsal, ventral, medial and lateral regions. **(B)** Representative toluidine blue-stained femoral cortical sections from rabbits at 1, 2 and 3 months of age, showing the transition from vascular canal-rich immature cortex to early osteonal organization. **(C)** Quantification of overall vascular canal density from 1 to 3 months of age, calculated as the animal-level mean across the four cortical regions. Vascular canal density increased from 1 to 2 months of age (P = 0.0407, Welch’s t-test). **(D)** Regional comparison of osteon area at 2 and 3 months of age across dorsal, ventral, medial and lateral cortical regions. P values for 2 versus 3 months were P = 8.14 × 10^-4, P = 2.78 × 10^-4, P = 0.0191 and P = 0.0030 for dorsal, ventral, medial and lateral regions, respectively, Welch’s t-test. Data are shown as mean ± SEM with individual animals overlaid. *P < 0.05, **P < 0.01 and ***P < 0.001.

Representative histological images showed that the 1-month-old cortex was characterized mainly by abundant vascular canals. Although immature matrix deposition was observed around some vascular canals, these structures did not meet the predefined criteria for typical osteons or Haversian systems. They were therefore classified as vascular canals rather than osteons. By 2 months of age, primary osteons became evident in the cortical bone. By 3 months, secondary osteons characterized by a central Haversian canal, concentric lamellae and a recognizable peripheral cement line became evident in some cortical regions (Figure 1B).

Quantitative analysis further showed age-dependent changes in vascular canal density from 1 to 3 months, indicating active organization and remodeling of the intracortical vascular canal system during this period (Figure 1C). Four-orientation 10x representative images and regional vascular canal density analysis supported this developmental pattern and revealed morphological heterogeneity among cortical regions. Therefore, the mean value across the four orientations was used as the animal-level measurement in subsequent main analyses to reduce the influence of regional variation (Supplementary Figures 1a,b).

Because typical osteons meeting the predefined criteria were not detected in 1-month-old samples, osteon area was quantified only in 2- and 3-month-old rabbits. The four-orientation analysis of osteon area from 2 to 3 months showed that identifiable osteons had formed during this period and could be used as a quantitative readout of early osteon morphological development (Figure 1D). Together, these findings demonstrate that the 1–3-month interval captures the onset and early progression of osteon formation, from a vascular canal-rich immature cortex at 1 month to evident primary and emerging secondary osteonal structures at 2 and 3 months. We therefore refer to this interval as an early osteon-forming window in the present study.

### AIMD induces gut microbiota disturbance during the early osteon-forming window

Based on this observed developmental progression, we next applied antibiotic-induced microbiota disturbance (AIMD) during this period to evaluate whether gut microbiota perturbation could affect subsequent osteon development. AIMD was initiated at 1 month of age and continued until sample collection at 3 months. At the endpoint, fecal samples were collected for 16S rRNA gene sequencing, while femoral cortical bone was collected for histology, micro-CT, metabolomics, RNA-seq and RT-qPCR analyses (Figure 2A).

**FIGURE 2:**
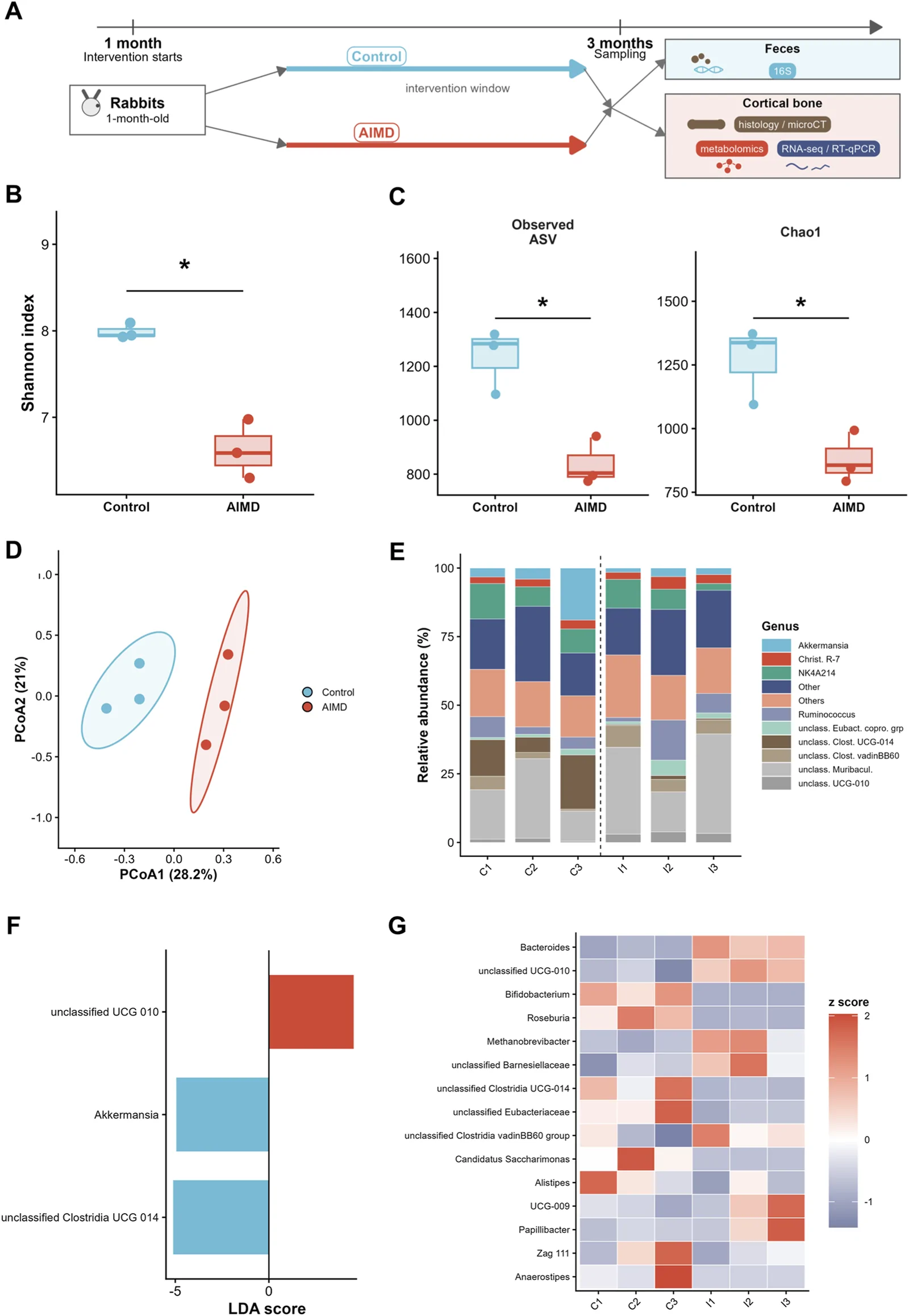
AIMD alters gut microbiota diversity and community composition. **(A)** Experimental design of antibiotic-induced microbiota disturbance (AIMD) and multi-omics sampling. **(B,C)** Alpha-diversity analysis showing Shannon index **(B)** and observed ASVs/Chao1 richness indices **(C)** in Control and AIMD groups. P values were P = 0.0151 for Shannon index, P = 0.0106 for observed ASVs and P = 0.0233 for Chao1, Welch’s t-test. **(D)** Bray-Curtis principal coordinate analysis (PCoA) showing gut microbial community structure between groups. Bray-Curtis dissimilarity was evaluated using permutational multivariate analysis of variance (PERMANOVA), with dispersion assessed by betadisper. **(E)** Genus-level microbial composition in individual samples. **(F)** Linear discriminant analysis effect size (LEfSe) analysis showing discriminatory bacterial taxa ranked by linear discriminant analysis (LDA) score. **(G)** Z-score heatmap of representative differential taxa between Control and AIMD groups. Data in B and C are shown as mean ± SEM with individual samples overlaid. *P < 0.05.

16S rRNA gene sequencing showed that AIMD markedly reduced gut microbial alpha diversity. Compared with controls, the AIMD group showed a lower Shannon index, indicating reduced microbial diversity and evenness (Figure 2B). Richness-related indices, including observed ASVs and Chao1, were also lower in the AIMD group, suggesting a reduction in detectable microbial features and species richness (Figure 2C). These results indicate that antibiotic treatment effectively induced gut microbiota disturbance during this early postnatal developmental period.

Beta diversity analysis further showed a separation tendency between the Control and AIMD groups in Bray-Curtis PCoA space, indicating that AIMD altered the overall structure of the gut microbial community (Figure 2D). PERMANOVA showed that group assignment accounted for part of the Bray-Curtis dissimilarity, while betadisper analysis was used to evaluate whether within-group dispersion contributed to this pattern. Thus, AIMD not only reduced alpha diversity but also reshaped the overall microbial community structure.

At the genus level, Control and AIMD samples displayed distinct compositional patterns, consistent with a shift in dominant microbial taxa after antibiotic treatment (Figure 2E). LEfSe analysis identified several group-associated taxa, including Control-enriched *Akkermansia* and Clostridia UCG-014, and AIMD-enriched UCG-010 (Figure 2F). A heatmap of top differential taxa further showed coordinated group-related patterns, with *Bacteroides* relatively enriched in the AIMD group and *Bifidobacterium*, *Roseburia*, *Alistipes* and *Anaerostipes* relatively enriched in the Control group (Figure 2G).

Together, these data show that AIMD effectively induced gut microbiota disturbance during the 1–3-month early osteon-forming window, characterized by reduced alpha diversity, altered beta diversity and reshaped genus-level composition. Given the limited sample size, the LEfSe and differential-taxa heatmap results should be interpreted as exploratory microbial features. These microbiota data provide the upstream perturbation framework for subsequent analyses of AIMD-associated changes in osteon morphology, cortical bone metabolism and cortical bone transcriptional programs.

### AIMD alters osteon morphology and compromises cortical bone mechanical performance

After confirming that AIMD effectively perturbed the gut microbiota, we next examined whether this intervention affected osteon morphology in 3-month-old rabbit cortical bone. Histological observation showed that the cortical bone of control rabbits contained organized osteonal structures, including primary osteons and emerging secondary osteons (Figure 3A). In contrast, AIMD-treated rabbits displayed less organized osteonal architecture, characterized by fewer secondary osteons and reduced regularity of osteon morphology (Figure 3B). Representative histological images from the four predefined cortical regions further indicated that AIMD-associated osteon alterations were not restricted to a single cortical sampling region (Supplementary Figure 2a).

**FIGURE 3:**
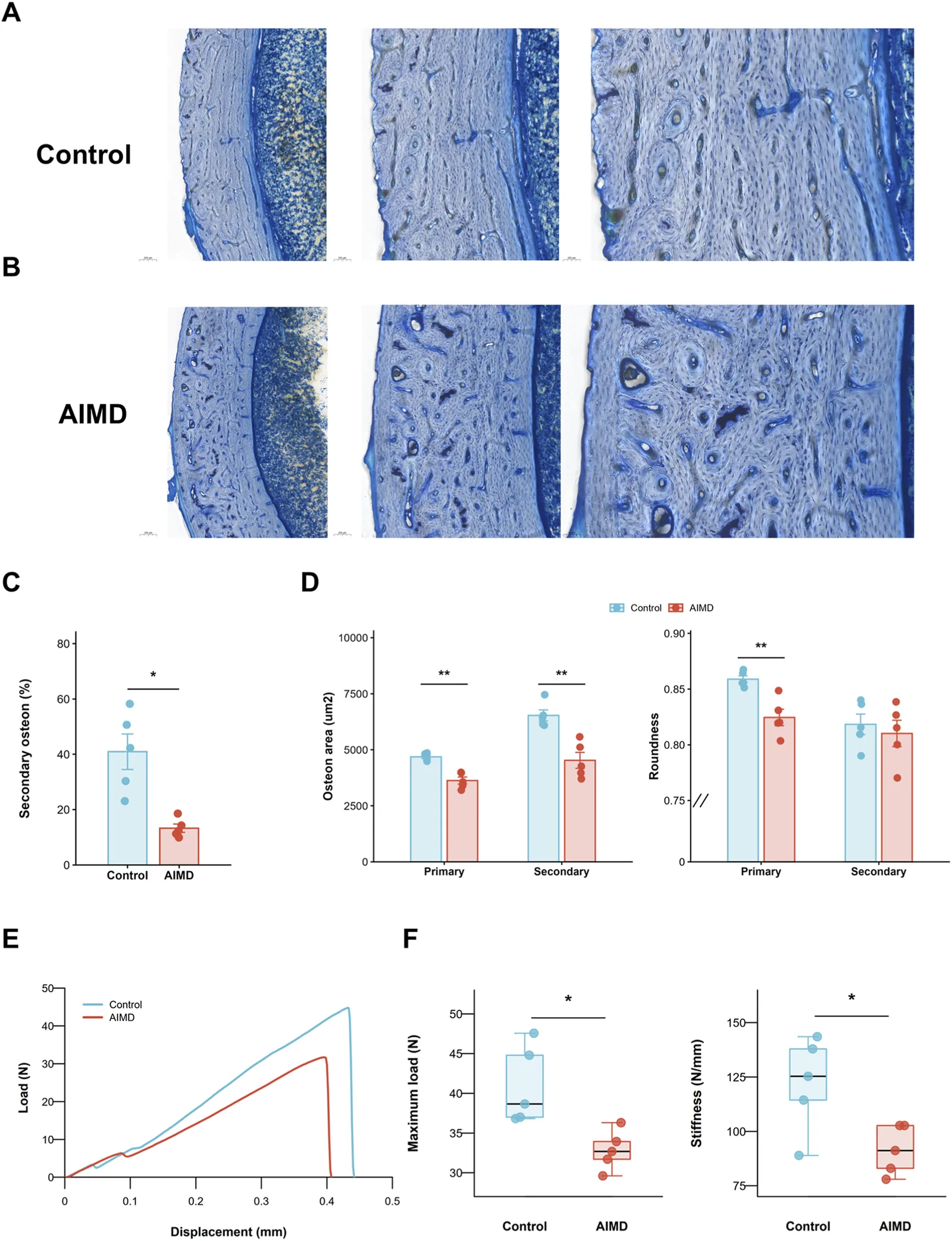
AIMD impairs osteon-level cortical bone organization and mechanical performance. **(A,B)** Representative femoral cortical bone histological images from Control **(A)** and AIMD **(B)** rabbits at 3 months of age, showing osteonal organization at 5×, ×10 and ×20 digital magnifications. **(C)** Quantification of secondary osteon percentage among identifiable osteonal structures (P = 0.0112, Welch’s t-test). **(D)** Quantitative comparison of primary and secondary osteon morphology, including osteon area and roundness. Significant P values were P = 0.00113 for primary osteon area, P = 0.00223 for secondary osteon area and P = 0.00695 for primary osteon roundness, Welch’s t-test. **(E)** Representative force-displacement curves from the Control and AIMD groups during three-point bending. **(F)** Comparison of maximum load and stiffness between the Control and AIMD groups. Box plots show the median and interquartile range, and individual points represent independent animals (n = 5 per group). Welch’s t-test yielded P = 0.0167 for maximum load and P = 0.0313 for stiffness. *P < 0.05 and **P < 0.01.

To quantitatively assess the effect of AIMD on osteon maturation, we first compared the proportion of secondary osteons among all identifiable osteons. The AIMD group showed a lower secondary osteon percentage than the control group, suggesting impaired progression toward more mature secondary osteonal organization (Figure 3C). We then analyzed morphological parameters separately for primary and secondary osteons. AIMD was associated with smaller primary and secondary osteon areas and reduced osteon roundness, with the change in roundness being more evident in primary osteons (Figure 3D). Additional osteon morphometric parameters, excluding osteon count, are shown in the supplementary figure (Supplementary Figure 2b).

We further used micro-CT to evaluate whether AIMD caused detectable changes in gross cortical bone structure. Longitudinal sections, cross-sections and 3D cortical bone reconstructions did not reveal obvious structural differences between Control and AIMD rabbits (Supplementary Figures 3a,b). Quantitative micro-CT analysis likewise showed no significant differences in conventional cortical parameters, including cortical thickness, cortical area and tissue mineral density (Supplementary Figures 3c–e). These results indicate that, at 3 months of age, conventional micro-CT measurements did not capture marked AIMD-associated changes in overall cortical geometry or mineralization.

Despite the absence of significant changes in conventional micro-CT cortical parameters, three-point bending tests revealed reduced mechanical performance in AIMD-treated cortical bone. Representative force-displacement curves showed a lower load-bearing response in the AIMD group than in controls (Figure 3E). Quantitative analysis further showed significantly lower maximum load and stiffness in the AIMD group, indicating impaired cortical bone mechanical properties after AIMD intervention (Figure 3F).

Together, these findings show that AIMD altered osteon morphology and reduced cortical bone mechanical performance in 3-month-old rabbits. The discrepancy between unchanged gross micro-CT parameters and impaired mechanical behavior raises the possibility that alterations in osteon microarchitecture and other tissue-material properties not captured by conventional micro-CT may contribute to the observed mechanical phenotype.

### AIMD is associated with femoral metabolomic remodeling dominated by lipid-related metabolites

After observing AIMD-associated changes in osteon morphology and cortical bone mechanical performance, we performed untargeted metabolomic profiling of femoral tissue to determine whether gut microbiota disturbance was accompanied by changes in the local bone metabolic environment. PCA showed a partial separation between Control and AIMD samples, indicating that AIMD was associated with a shift in the overall femoral metabolomic profile (Figure 4A). A supervised score plot showed a similar group-separation pattern and was used as an additional visualization of group-level metabolic differences (Figure 4B).

**FIGURE 4:**
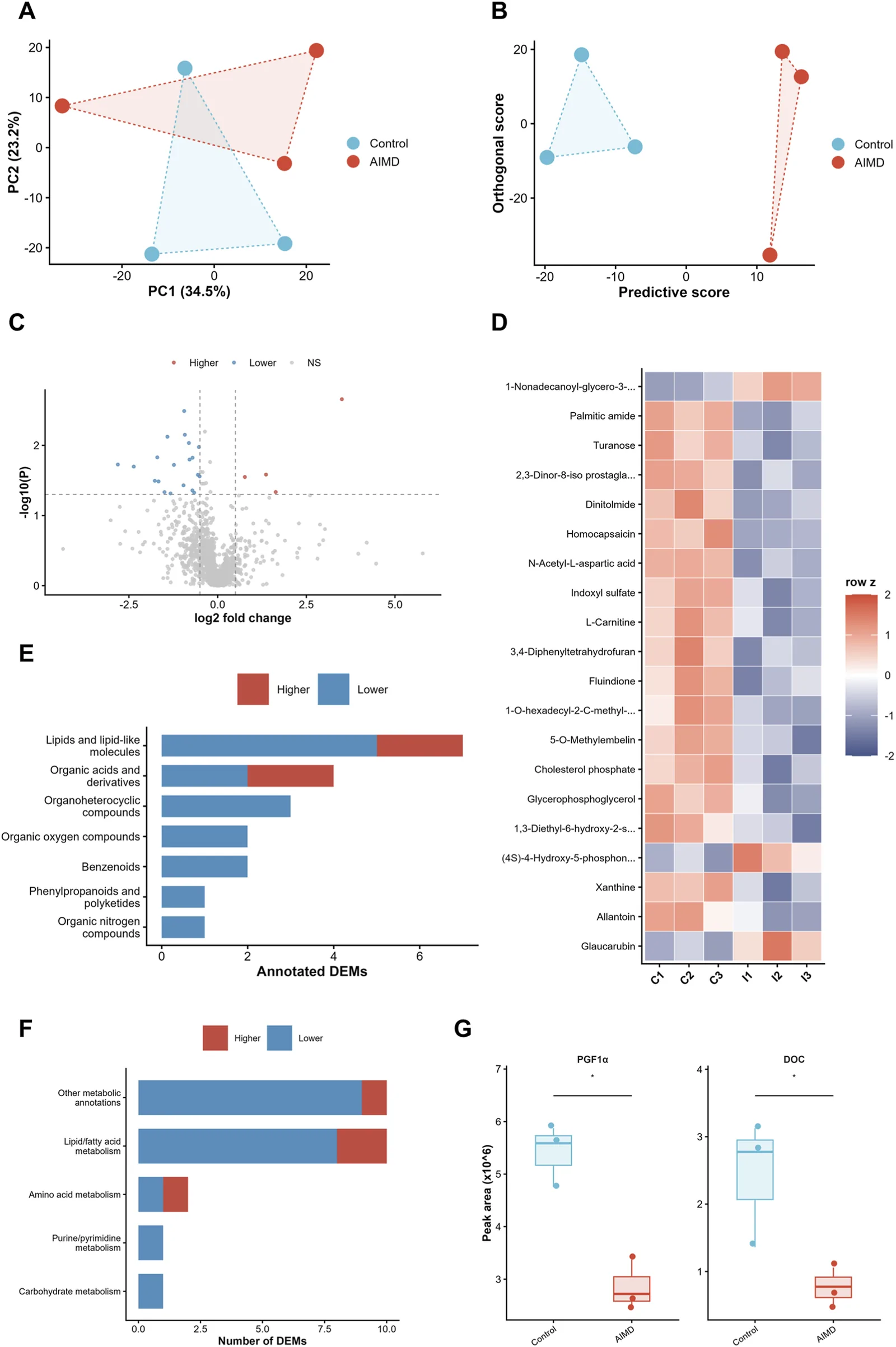
AIMD is associated with local femoral metabolic remodeling. **(A)** Principal component analysis (PCA) of femoral cortical tissue metabolomic profiles from Control and AIMD groups. **(B)** Supervised score plot showing group separation in the femoral metabolomic dataset. **(C)** Volcano plot of differential metabolites between Control and AIMD groups. **(D)** Heatmap of representative top differential metabolites, shown as row-scaled z-scores. **(E)** Classification of differential metabolites according to database or superclass annotation. **(F)** Distribution of differential metabolites across metabolism-related categories. **(G)** Peak-area comparison of representative metabolites PGF1α and DOC. P values were P = 0.0071 for PGF1α and P = 0.0327 for DOC, Welch’s t-test. Data in G are shown as mean ± SEM with individual samples overlaid. *P < 0.05 and **P < 0.01.

Differential metabolite analysis identified a set of metabolites that were increased or decreased in AIMD-treated rabbits compared with controls (Figure 4C). A heatmap of top differential metabolites showed relatively consistent group-related clustering patterns, further supporting AIMD-associated remodeling of the femoral metabolic profile (Figure 4D).

Classification of differential metabolites showed that these changes involved multiple chemical and metabolic categories, with a notable contribution from lipids and lipid-like molecules, organic acids and derivatives, amino acid-related metabolites and other small molecules (Figure 4E). Further metabolism-type classification indicated that lipid/fatty acid-related metabolites represented a major component of the AIMD-associated metabolic changes, and most of these lipid-related differential metabolites were reduced in the AIMD group (Figure 4F). These findings suggest that AIMD altered the local femoral metabolic environment, with prominent involvement of lipid-related metabolic remodeling.

Among representative differential metabolites, PGF1α and deoxycorticosterone (DOC) were both lower in AIMD-treated samples (Figure 4G). PGF1α is a prostaglandin-related lipid mediator, whereas DOC is a steroid hormone-related metabolite. Their reduction indicates that AIMD-associated femoral metabolic changes involved lipid mediator-related and steroid-related metabolic features. Together, these data suggest that AIMD reshaped the femoral metabolic microenvironment, with lipid-related metabolic disturbance as a major feature.

### AIMD is accompanied by cortical bone transcriptional remodeling during early osteonal organization

After identifying AIMD-associated remodeling of the femoral metabolic profile, we next performed RNA-seq analysis of cortical bone to determine whether gut microbiota disturbance was accompanied by local transcriptional changes during early osteonal organization. PCA showed partial separation between Control and AIMD samples, indicating that AIMD was associated with a shift in the overall cortical bone transcriptomic profile (Figure 5A). Consistently, hierarchical clustering of all DEGs separated the AIMD samples from the Control samples and revealed distinct group-associated expression patterns (Figure 5B). Volcano-style differential expression analysis further identified a set of genes that were upregulated or downregulated in AIMD-treated rabbits compared with controls (Figure 5C).

**FIGURE 5:**
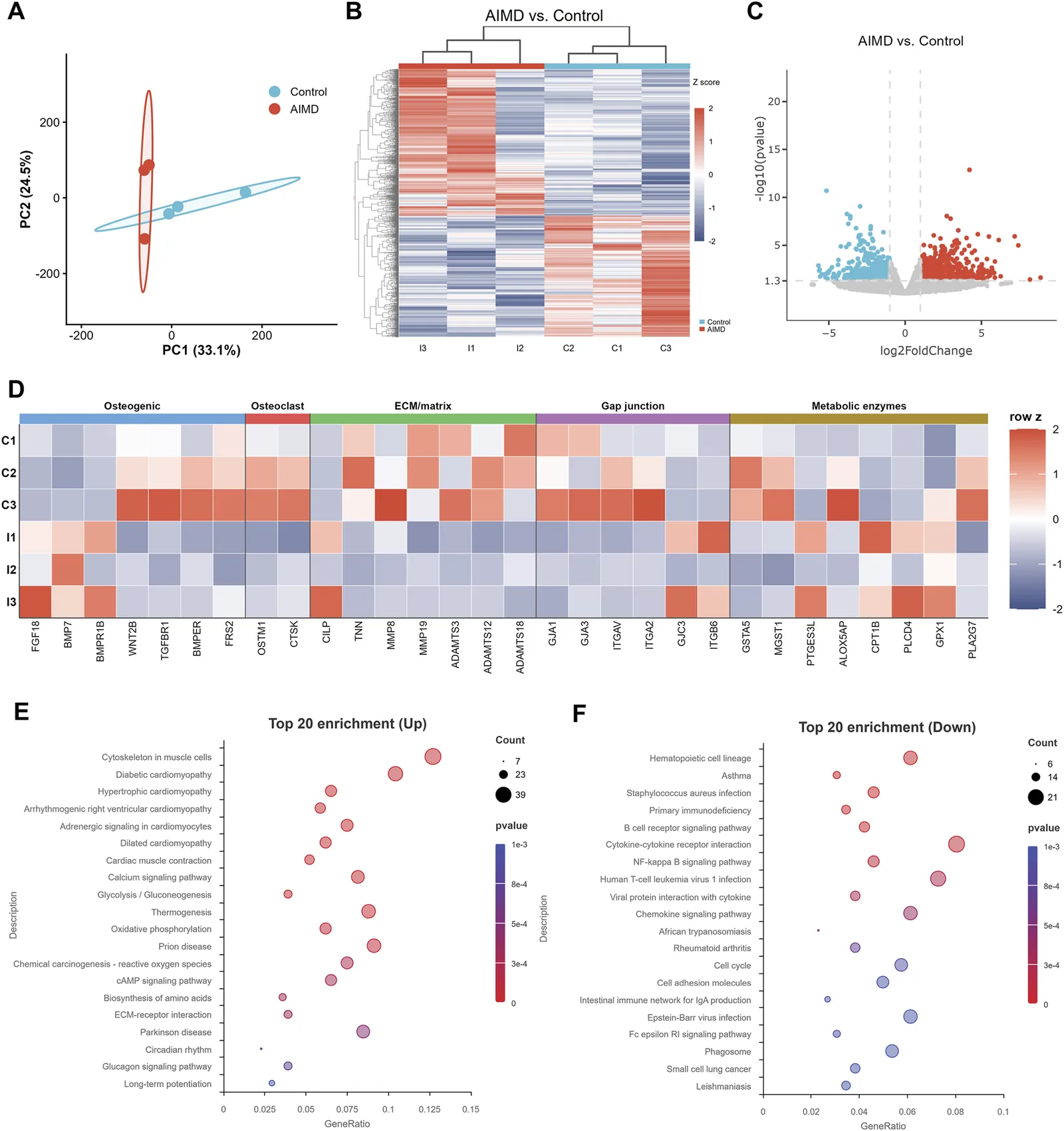
AIMD is associated with cortical bone transcriptional remodeling. **(A)** PCA of cortical bone RNA-seq profiles from Control and AIMD groups. **(B)** Hierarchical clustering heatmap of all differentially expressed genes (DEGs) between Control and AIMD cortical bone samples. Rows represent DEGs and columns represent individual samples. Expression values are row-scaled z-scores. **(C)** Differential gene-expression plot showing upregulated and downregulated genes in AIMD versus Control cortical bone. **(D)** RT-qPCR heatmap of selected bone-related and metabolism-related genes, including osteogenesis/bone-development signaling, osteoclast-related genes, extracellular matrix (ECM) /bone matrix genes, osteocyte communication/gap-junction genes and metabolic enzyme genes. **(E, F)** Kyoto Encyclopedia of Genes and Genomes (KEGG) enrichment analysis of upregulated **(E)** and downregulated **(F)** DEGs. Differential expression was analyzed from the gene count matrix using DESeq2, with DEGs defined as P < 0.05 and |log2 fold change| > 1.

To focus on transcriptional programs related to osteon development and bone remodeling, we next examined representative genes by RT-qPCR. These genes covered five functional modules: osteogenesis/bone-development signaling, osteoclast-related genes, extracellular matrix (ECM)/bone matrix, osteocyte communication/gap junction, and metabolic enzymes. The RT-qPCR heatmap showed group-associated expression patterns across these modules, supporting AIMD-associated changes in bone matrix organization, remodeling activity, osteocyte communication and metabolic-enzyme-related transcriptional programs (Figure 5D).

KEGG enrichment analysis of upregulated DEGs indicated that AIMD-associated transcriptional changes involved pathways related to cytoskeletal organization, calcium signaling, contractility-related processes and energy metabolism (Figure 5E). In contrast, downregulated DEGs were associated with pathways related to immune/inflammatory responses, gap junction and cell-cell communication, cadherin signaling, glutathione metabolism and xenobiotic/drug-metabolism-related processes (Figure 5F). These enrichment patterns suggest that AIMD induced broad cortical bone transcriptional remodeling rather than alteration of a single isolated pathway.

Together, the cortical bone transcriptomic results indicate that AIMD-associated osteon morphological changes were accompanied by altered transcriptional programs related to bone-development signaling, matrix remodeling, osteocyte communication and metabolic regulation. These findings provide molecular context for AIMD-associated local femoral metabolic and transcriptional remodeling accompanying impaired early osteonal organization.

### Integrated transcriptomic-metabolomic analysis identifies molecular associations after AIMD

To explore the relationship between AIMD-associated transcriptional and metabolic changes, we performed Pearson correlation analysis between the top 100 named DEGs and all differential metabolites. The correlation heatmap showed positive and negative associations between differential genes and metabolites (Figure 6A), suggesting that transcriptional and metabolic changes may be related.

**FIGURE 6:**
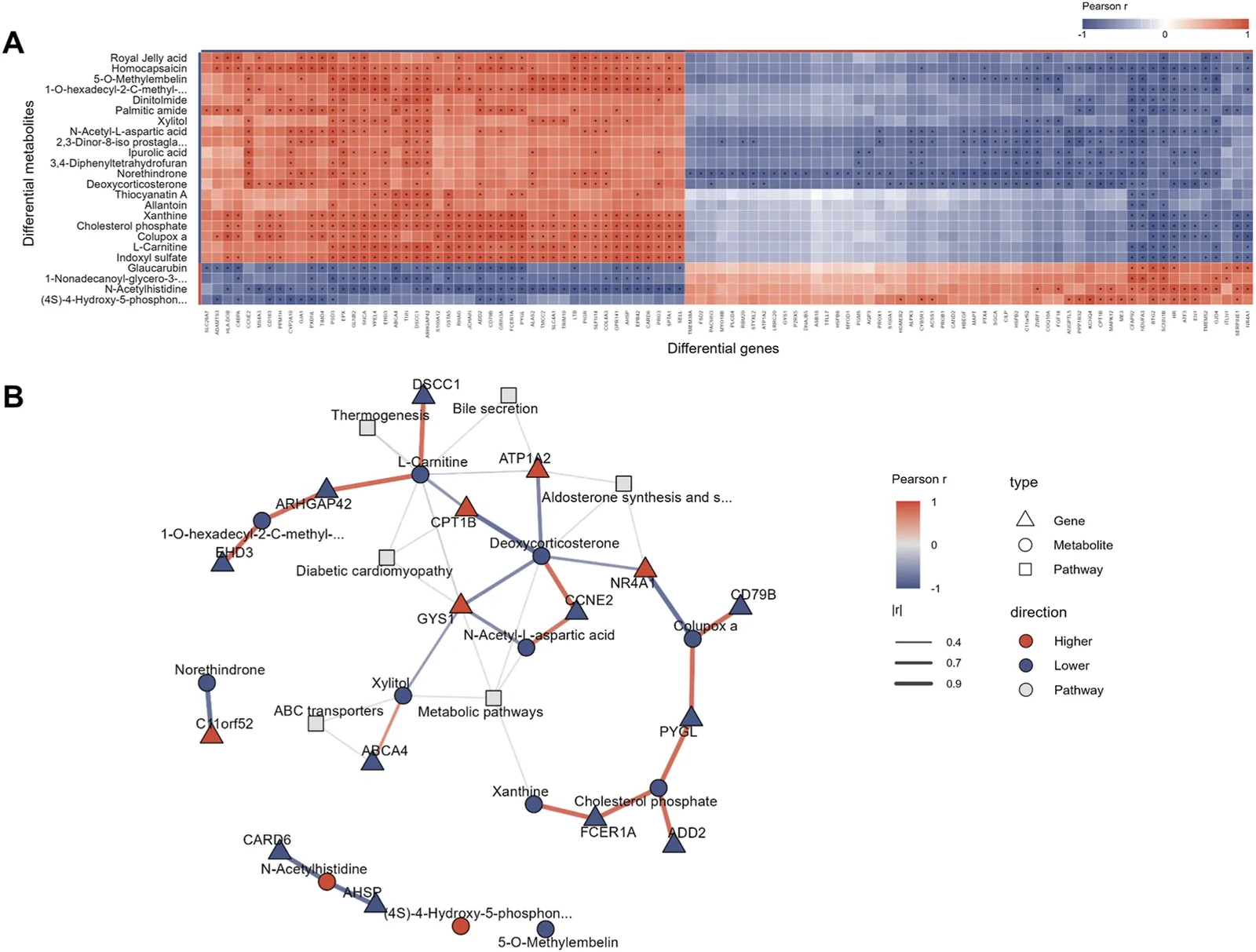
Integrated transcriptome-metabolome analysis links altered metabolites with cortical bone gene-expression programs. **(A)** Pearson correlation heatmap between differential metabolites and the top 100 named differentially expressed genes. Rows represent differential metabolites, and columns represent differentially expressed genes. Gene expression was based on log2(FPKM + 1), and metabolite abundance was based on log2 (peak area + 1). **(B)** KGML-supported gene-metabolite-pathway network connecting selected differential metabolites, differentially expressed genes and shared metabolic pathways. Only genes with annotated gene symbols were retained for the integrative analysis. *P < 0.05, **P < 0.01 and ***P < 0.001.

We further constructed a KEGG/KGML-supported gene-metabolite-pathway network based on these differential genes and metabolites. The network highlighted several connected gene-metabolite modules involving metabolic regulation, metabolite transport and steroid-related metabolic features (Figure 6B). Representative links included CPT1B with L-carnitine, and DOC-related connections with transcriptional regulators and metabolic genes.

Together, this integrated analysis indicates that AIMD-associated osteon alterations were accompanied by transcriptomic and metabolomic changes in cortical bone. These results provide an exploratory molecular framework for linking gut microbiota disturbance to local bone metabolic and transcriptional remodeling.

## Discussion

In this study, we characterized cortical canal remodeling and early osteonal organization across the first three postnatal months in New Zealand rabbits, and used this developmental period to examine how AIMD is associated with cortical bone maturation. AIMD effectively disrupted the gut microbial community, as reflected by reduced microbial diversity and altered community structure, and was accompanied by impaired osteonal organization at 3 months of age. Histological analyses showed a lower proportion of secondary osteons and altered osteon area and roundness in AIMD-treated rabbits, whereas conventional micro-CT parameters of cortical bone geometry and mineral density were relatively unchanged. In parallel, three-point bending tests indicated reduced cortical bone mechanical performance, suggesting that the skeletal effects of AIMD were more evident at the level of osteon organization and tissue quality than at the level of bulk cortical structure. Untargeted femoral metabolomics and cortical-bone transcriptomic analyses further revealed local metabolic and transcriptional remodeling, particularly involving lipid-related metabolites, extracellular matrix organization, osteocyte communication and metabolic enzyme programs. Together, these findings suggest that early gut microbiota disturbance is associated with impaired osteon-level cortical bone maturation and reduced mechanical competence, accompanied by remodeling of the local molecular environment.

The developmental context of this study is important for interpreting these findings. Rather than treating the rabbit femoral cortex as a static structure, we first characterized a period during which cortical canals and osteonal structures undergo active postnatal organization. At 1 month of age, the cortex was dominated by vascular canals and immature canal-associated matrix deposition, whereas typical osteonal structures were not yet clearly established. By 2 months, primary osteons became more evident, and by 3 months, more organized osteonal structures, including secondary osteons, began to emerge. This developmental pattern is consistent with the active organization of the intracortical vascular and osteonal systems previously described in rabbit long bones (Pazzaglia et al., 2008). Accordingly, the 1–3-month interval examined here captures the onset and early progression of osteon formation and is therefore referred to as an early osteon-forming window in the present study. Characterizing this developmental progression was important because the effect of microbiota disturbance would be difficult to interpret without knowing whether osteon formation was actively occurring. The use of predefined dorsal, ventral, medial and lateral cortical regions also helped account for regional heterogeneity within the cortex, allowing subsequent analyses to focus on animal-level osteonal maturation rather than isolated microscopic fields.

A further implication of this work is that the gut microbiota–bone axis should not be considered only through conventional measures of bone mass or trabecular microarchitecture. Previous studies have commonly evaluated microbiota-related skeletal effects using outcomes such as BMD, trabecular bone parameters, bone turnover markers or whole-bone mechanical properties (Lyu et al., 2023; Yan et al., 2016; Guss et al., 2017). Notably, several microbiota-related intervention studies have reported detectable skeletal or trabecular changes without corresponding alterations in conventional cortical micro-CT indices (Hathaway-Schrader et al., 2019; Carson et al., 2023; Rios-Arce et al., 2020), and impaired bone tissue strength has also been observed in the absence of noticeable changes in cortical bone geometry (Castaneda et al., 2021). These findings suggest that conventional measurements of cortical geometry and mineralization, although important, may not fully capture changes in cortical bone tissue organization, particularly during growth. Osteons are fundamental structural units of cortical bone that integrate vascular canals, matrix deposition, remodeling activity and local tissue organization. Changes in osteon morphology may therefore provide information about cortical bone quality that is not apparent from bulk micro-CT measurements alone. By focusing on primary and secondary osteonal structures, this study extends the gut microbiota–bone framework to the level of cortical osteon organization. This perspective is particularly relevant during development, when cortical bone is undergoing active canal remodeling and osteonal maturation, and when perturbations in the systemic or metabolic environment may influence the establishment of tissue-level skeletal quality.

The dissociation between conventional micro-CT parameters and mechanical performance is also informative. In the present study, AIMD did not produce marked changes in cortical thickness, cortical area or tissue mineral density at 3 months of age, yet osteon morphology and bending performance were altered. Given the absence of marked changes in conventional cortical micro-CT parameters, the concurrent alterations in osteonal organization and mechanical performance raise the possibility that osteon-level organization contributes to the mechanical phenotype after AIMD, although a causal relationship cannot be established from the present data. Cortical bone strength is determined not only by bone size and mineral content, but also by cortical microstructure and local material properties (Augat and Schorlemmer, 2006). At the osteon level, lamellar organization, osteonal boundaries and tissue-level mechanical heterogeneity contribute to crack resistance and cortical toughness (Katsame et al., 2015; Yin et al., 2021). Accordingly, the altered osteonal organization observed here represents one plausible contributor to the reduced mechanical performance. In this context, the reduced maximum load and stiffness observed after AIMD may reflect impaired cortical tissue quality rather than a simple loss of cortical bone mass. These findings highlight the value of osteon-level histological assessment as a complement to micro-CT for evaluating cortical bone quality during growth.

The metabolomic findings provide one possible molecular context for these osteon-level changes. AIMD was associated with a shift in the femoral cortical bone metabolic profile, and several differential metabolites were related to lipid-mediated or steroid-related metabolism. Among the representative metabolites, PGF1α reflects a prostaglandin-related lipid mediator, whereas DOC is linked to steroid hormone metabolism. Although these metabolites cannot by themselves define a causal pathway, their alteration suggests that AIMD may reshape the local metabolic environment required for coordinated cortical bone remodeling. Lipids and their bioactive derivatives, including prostaglandins, can regulate bone-cell activity, inflammatory signaling and tissue remodeling (During et al., 2015). The observed metabolic changes may therefore reflect an altered local biochemical environment during osteonal maturation. However, because untargeted metabolomics does not establish pathway activity or causality, the functional relevance of PGF1α, DOC and related metabolites requires targeted validation.

The transcriptomic findings further support local molecular remodeling after AIMD. Genes related to ECM organization may influence matrix deposition and tissue architecture, whereas alterations in gap-junction and osteocyte-communication programs may affect the coordination of bone cells within the cortical network (Zappalà et al., 2023; Zhao et al., 2023). Together with changes in metabolic enzyme genes, these findings indicate that AIMD was associated with coordinated remodeling of cellular programs relevant to osteonal organization, although the small sample size and bulk-tissue design preclude attribution to specific cell populations or pathways.

Taken together, these findings support an exploratory conceptual model, proposed as a hypothesis for future testing, in which AIMD-induced gut microbiota disturbance is accompanied by local metabolic and transcriptional remodeling in developing cortical bone, which may contribute to impaired osteonal organization and reduced mechanical performance. Within this proposed model, we hypothesize that microbiota-related metabolic signaling may influence cortical-bone transcriptional programs, including metabolic enzyme-related genes, thereby contributing to local metabolic remodeling during osteonal maturation. These changes are accompanied by transcriptional alterations in pathways related to bone-development signaling, ECM organization and osteocyte communication. The observed changes in immune/inflammatory-related transcriptional programs suggest that immune regulation may also contribute to this response, although its cellular basis and relative contribution remain to be determined. The convergence of these omics-level changes with histological and mechanical phenotypes suggests that osteonal maturation may be sensitive to the metabolic and molecular state of the cortical bone microenvironment. This exploratory model provides a testable framework for future studies to examine the temporal and causal relationships among microbiota disturbance, local metabolic and transcriptional changes, osteonal organization and cortical bone strength.

From a translational perspective, an important implication of this study is that osteonal organization may represent a dynamic and biologically responsive component of cortical bone quality. The present findings show that osteonal maturation can be altered in the setting of an experimental systemic perturbation during skeletal growth, supporting the concept that osteon-level organization is not merely a static histological feature but may be responsive to changes in the biological environment. This osteon-centered perspective may have broader relevance to conditions in which cortical bone quality depends on osteonal remodeling or re-establishment, including osteoporosis and fracture repair. However, whether microbiota-related regulation contributes to osteonal remodeling in these pathological or regenerative settings remains to be determined.

Several limitations should be acknowledged. First, although the bone morphometric and mechanical analyses were performed at the animal level, the matched multi-omics analyses were limited by the small number of samples, and the transcriptome-metabolome correlations should therefore be interpreted as exploratory rather than definitive. Second, the AIMD regimen was designed to primarily perturb the intestinal microbial community while limiting direct systemic antibiotic exposure, as rifaximin and neomycin are minimally absorbed from the gastrointestinal tract. This pharmacological design strengthens the interpretation that the observed skeletal phenotype was predominantly associated with microbiota disturbance. However, complete separation of microbiota-mediated and antibiotic-specific effects is not possible, particularly because metronidazole is systemically absorbed. Future microbiota-restoration or transplantation experiments will therefore be needed to confirm causality. Although no overt differences in spontaneous activity were observed during routine monitoring, locomotor activity was not quantitatively assessed, and subtle activity-related effects on cortical bone cannot be completely excluded. Third, this study focused on endpoint measurements at 3 months of age, which limits the ability to determine the temporal sequence linking microbiota disturbance, metabolic remodeling, transcriptional changes and osteonal maturation. Fourth, the untargeted metabolomics results identify candidate metabolites such as PGF1α and DOC, but targeted validation and functional testing are required to confirm their biological relevance. Finally, the classification of primary and secondary osteonal structures was based mainly on histological morphology; future studies combining dynamic bone labeling, higher-resolution three-dimensional imaging and cell-specific molecular assays would further strengthen the interpretation of osteonal remodeling. Despite these limitations, this study establishes a useful experimental framework linking early gut microbiota disturbance to cortical osteon-level organization, local molecular remodeling and bone mechanical competence during growth.

## Data Availability

The datasets presented in this study can be found in online repositories. The names of the repository/repositories and accession number(s) can be found below: https://www.ncbi.nlm.nih.gov/, BioProject: PRJNA1492190.

## References

[B1] Augat P. Schorlemmer S. (2006). The role of cortical bone and its microstructure in bone strength. Age Ageing 35 (Suppl. 2), ii27–ii31. 10.1093/ageing/afl081 16926200

[B2] Bokulich N. A. Kaehler B. D. Rideout J. R. Dillon M. Bolyen E. Knight R. (2018). Optimizing taxonomic classification of marker-gene amplicon sequences with QIIME 2's q2-feature-classifier plugin. Microbiome 6 (1), 90. 10.1186/s40168-018-0470-z 29773078 PMC5956843

[B3] Bolyen E. Rideout J. R. Dillon M. R. Bokulich N. A. Abnet C. C. Al-Ghalith G. A. (2019). Reproducible, interactive, scalable and extensible microbiome data science using QIIME 2. Nat. Biotechnol. 37 (8), 852–857. 10.1038/s41587-019-0209-9 31341288 PMC7015180

[B4] Callahan B. J. McMurdie P. J. Rosen M. J. Han A. W. Johnson A. J. Holmes S. P. (2016). DADA2: High-resolution sample inference from illumina amplicon data. Nat. Methods 13 (7), 581–583. 10.1038/nmeth.3869 27214047 PMC4927377

[B5] Carson M. D. Warner A. J. Hathaway-Schrader J. D. Geiser V. L. Kim J. Gerasco J. E. (2023). Minocycline-induced disruption of the intestinal FXR/FGF15 axis impairs osteogenesis in mice. JCI Insight 8 (1), e160578. 10.1172/jci.insight.160578 36413391 PMC9870091

[B6] Castaneda M. Smith K. M. Nixon J. C. Hernandez C. J. Rowan S. (2021). Alterations to the gut microbiome impair bone tissue strength in aged mice. Bone Rep. 14, 101065. 10.1016/j.bonr.2021.101065 33937443 PMC8079457

[B7] Chang B. Liu X. (2022). Osteon: structure, turnover, and regeneration. Tissue Eng. Part B Rev. 28 (2), 261–278. 10.1089/ten.teb.2020.0322 33487116 PMC9063188

[B8] Cunningham F. Allen J. E. Allen J. Alvarez-Jarreta J. Amode M. R. Armean I. M. (2022). Ensembl 2022. Nucleic Acids Res. 50 (D1), D988. 10.1093/nar/gkab1049 34791404 PMC8728283

[B9] During A. Penel G. Hardouin P. (2015). Understanding the local actions of lipids in bone physiology. Prog. Lipid Res. 59, 126–146. 10.1016/j.plipres.2015.06.002 26118851

[B10] Guss J. D. Horsfield M. W. Fontenele F. F. Sandoval T. N. Luna M. Apoorva F. (2017). Alterations to the gut microbiome impair bone strength and tissue material properties. J. Bone Min. Res. 32 (6), 1343–1353. 10.1002/jbmr.3114 28244143 PMC5466506

[B11] Harrison K. D. Sales E. Hiebert B. D. Panahifar A. Zhu N. Arnason T. (2022). Direct assessment of rabbit cortical bone basic multicellular unit longitudinal erosion rate: a 4D synchrotron-based approach. J. Bone Min. Res. 37 (11), 2244–2258. 10.1002/jbmr.4700 36069373 PMC10091719

[B12] Hathaway-Schrader J. D. Steinkamp H. M. Chavez M. B. Poulides N. A. Kirkpatrick J. E. Chew M. E. (2019). Antibiotic perturbation of gut microbiota dysregulates osteoimmune cross talk in postpubertal skeletal development. Am. J. Pathol. 189 (2), 370–390. 10.1016/j.ajpath.2018.10.017 30660331 PMC6360355

[B13] Isaksson H. Harjula T. Koistinen A. Iivarinen J. Seppänen K. Arokoski J. P. (2010). Collagen and mineral deposition in rabbit cortical bone during maturation and growth: effects on tissue properties. J. Orthop. Res. 28 (12), 1626–1633. 10.1002/jor.21186 20540098

[B14] Isojima T. Sims N. A. (2021). Cortical bone development, maintenance and porosity: genetic alterations in humans and mice influencing chondrocytes, osteoclasts, osteoblasts and osteocytes. Cell. Mol. Life Sci. 78 (15), 5755–5773. 10.1007/s00018-021-03884-w 34196732 PMC11073036

[B15] Kanehisa M. Goto S. (2000). KEGG: kyoto encyclopedia of genes and genomes. Nucleic Acids Res. 28 (1), 27–30. 10.1093/nar/28.1.27 10592173 PMC102409

[B16] Katsamenis O. L. Jenkins T. Thurner P. J. (2015). Toughness and damage susceptibility in human cortical bone is proportional to mechanical inhomogeneity at the osteonal-level. Bone 76, 158–168. 10.1016/j.bone.2015.03.020 25863123

[B17] Kim D. Paggi J. M. Park C. Bennett C. Salzberg S. L. (2019). Graph-based genome alignment and genotyping with HISAT2 and HISAT-genotype. Nat. Biotechnol. 37 (8), 907–915. 10.1038/s41587-019-0201-4 31375807 PMC7605509

[B18] Koh N. Y. Y. Miszkiewicz J. J. Fac M. L. Wee N. K. Y. Sims N. A. (2024). Preclinical rodent models for human bone disease, including a focus on cortical bone. Endocr. Rev. 45 (4), 493–520. 10.1210/endrev/bnae004 38315213 PMC11244217

[B19] Liu C. Yang Y. N. Guo S. Cao Y. Wang L. N. Wu C. (2026). Gut microbiota-mediated regulation of skeletal development: a review of mechanistic analysis and interventional strategies. J. Adv. Res. 82, 397–410. 10.1016/j.jare.2025.07.033 40691987 PMC13001057

[B20] Livak K. J. Schmittgen T. D. (2001). Analysis of relative gene expression data using real-time quantitative PCR and the 2(-Delta Delta C(T)) method. Methods 25 (4), 402–408. 10.1006/meth.2001.1262 11846609

[B21] Love M. I. Huber W. Anders S. (2014). Moderated estimation of fold change and dispersion for RNA-seq data with DESeq2. Genome Biol. 15 (12), 550. 10.1186/s13059-014-0550-8 25516281 PMC4302049

[B22] Lyu Z. Hu Y. Guo Y. Liu D. (2023). Modulation of bone remodeling by the gut microbiota: a new therapy for osteoporosis. Bone Res. 11 (1), 31. 10.1038/s41413-023-00264-x 37296111 PMC10256815

[B23] Morgan E. F. Unnikrisnan G. U. Hussein A. I. (2018). Bone mechanical properties in healthy and diseased states. Annu. Rev. Biomed. Eng. 20, 119–143. 10.1146/annurev-bioeng-062117-121139 29865872 PMC6053074

[B24] Pazzaglia U. E. Bonaspetti G. Rodella L. F. Ranchetti F. Azzola F. (2007). Design, morphometry and development of the secondary osteonal system in the femoral shaft of the rabbit. J. Anat. 211 (3), 303–312. 10.1111/j.1469-7580.2007.00782.x 17848159 PMC2375807

[B25] Pazzaglia U. E. Bonaspetti G. Ranchetti F. Bettinsoli P. (2008). A model of the intracortical vascular system of long bones and of its organization: an experimental study in rabbit femur and tibia. J. Anat. 213 (2), 183–193. 10.1111/j.1469-7580.2008.00932.x 19172733 PMC2526107

[B26] Pazzaglia U. E. Zarattini G. Giacomini D. Rodella L. Menti A. M. Feltrin G. (2010). Morphometric analysis of the canal system of cortical bone: an experimental study in the rabbit femur carried out with standard histology and micro-CT. Anat. Histol. Embryol. 39 (1), 17–26. 10.1111/j.1439-0264.2009.00973.x 19874276

[B27] Peev S. Parushev I. Yotsova R. (2024). A modified protocol for staining of undecalcified bone samples using toluidine blue—A histological study in rabbit models. Appl. Sci. 14 (1), 461. 10.3390/app14010461

[B28] Poteres E. Hubert N. Poludasu S. Brigando G. Moore J. Keeler K. (2020). Selective regional alteration of the gut microbiota by diet and antibiotics. Front. Physiol. 11, 797. 10.3389/fphys.2020.00797 32733284 PMC7358400

[B29] Quast C. Pruesse E. Yilmaz P. Gerken J. Schweer T. Yarza P. (2013). The SILVA ribosomal RNA gene database project: improved data processing and web-based tools. Nucleic Acids Res. 41 (Database issue), D590–D596. 10.1093/nar/gks1219 23193283 PMC3531112

[B30] Rios-Arce N. D. Schepper J. D. Dagenais A. Schaefer L. Daly-Seiler C. S. Gardinier J. D. (2020). Post-antibiotic gut dysbiosis-induced trabecular bone loss is dependent on lymphocytes. Bone 134, 115269. 10.1016/j.bone.2020.115269 32061677 PMC7138712

[B31] Schneider C. A. Rasband W. S. Eliceiri K. W. (2012). NIH image to ImageJ: 25 years of image analysis. Nat. Methods 9 (7), 671–675. 10.1038/nmeth.2089 22930834 PMC5554542

[B32] Segata N. Izard J. Waldron L. Gevers D. Miropolsky L. Garrett W. S. (2011). Metagenomic biomarker discovery and explanation. Genome Biol. 12 (6), R60. 10.1186/gb-2011-12-6-r60 21702898 PMC3218848

[B33] Sjögren K. Engdahl C. Henning P. Lerner U. H. Tremaroli V. Lagerquist M. K. (2012). The gut microbiota regulates bone mass in mice. J. Bone Min. Res. 27 (6), 1357–1367. 10.1002/jbmr.1588 22407806 PMC3415623

[B34] Smith C. A. Want E. J. O'Maille G. Abagyan R. Siuzdak G. (2006). XCMS: processing mass spectrometry data for metabolite profiling using nonlinear peak alignment, matching, and identification. Anal. Chem. 78 (3), 779–787. 10.1021/ac051437y 16448051

[B35] Sud M. Fahy E. Cotter D. Brown A. Dennis E. A. Glass C. K. (2007). LMSD: LIPID MAPS structure database. Nucleic Acids Res. 35 (Database issue), D527–D532. 10.1093/nar/gkl838 17098933 PMC1669719

[B36] Tan J. Gong J. Liu F. Li B. Li Z. You J. (2022). Evaluation of an antibiotic cocktail for fecal microbiota transplantation in mouse. Front. Nutr. 9, 918098. 10.3389/fnut.2022.918098 35719145 PMC9204140

[B37] Wishart D. S. Guo A. Oler E. Wang F. Anjum A. Peters H. (2022). HMDB 5.0: the human metabolome database for 2022. Nucleic Acids Res. 50 (D1), D622. 10.1093/nar/gkab1062 34986597 PMC8728138

[B38] Yan J. Herzog J. W. Tsang K. Brennan C. A. Bower M. A. Garrett W. S. (2016). Gut microbiota induce IGF-1 and promote bone formation and growth. Proc. Natl. Acad. Sci. U. S. A. 113 (47), E7554. 10.1073/pnas.1607235113 27821775 PMC5127374

[B39] Yin D. Chen B. Lin S. (2021). Finite element analysis on multi-toughening mechanism of microstructure of osteon. J. Mech. Behav. Biomed. Mater 117, 104408. 10.1016/j.jmbbm.2021.104408 33657473

[B40] Zappalà A. Romano I. R. D'Angeli F. Musumeci G. Lo Furno D. Giuffrida R. (2023). Functional roles of connexins and gap junctions in osteo-chondral cellular components. Int. J. Mol. Sci. 24 (4), 4156. 10.3390/ijms24044156 36835567 PMC9967557

[B41] Zarrinpar A. Chaix A. Xu Z. Z. Chang M. W. Marotz C. A. Saghatelian A. (2018). Antibiotic-induced microbiome depletion alters metabolic homeostasis by affecting gut signaling and colonic metabolism. Nat. Commun. 9 (1), 2872. 10.1038/s41467-018-05336-9 30030441 PMC6054678

[B42] Zhao D. Wu J. Acosta F. M. Xu H. Jiang J. X. (2023). Connexin 43 hemichannels and prostaglandin E(2) release in anabolic function of the skeletal tissue to mechanical stimulation. Front. Cell. Dev. Biol. 11, 1151838. 10.3389/fcell.2023.1151838 37123401 PMC10133519

