# Supplementary material for: Gut microbiota disturbance disrupts osteonal organization and mechanical competence during postnatal cortical bone maturation in rabbits with local metabolic and transcriptional remodeling
Source: Front Cell Dev Biol. 2026 Oct 5;14:1937899. doi: 10.3389/fcell.2026.1937899 (PMC13677890; doi:10.3389/fcell.2026.1937899)
Supplement: Supplementary file 1 [file Supplementaryfile1.docx]

Supplementary Figure 1 | Regional vascular canal features during early postnatal cortical bone development. (a) Representative 10× histological images from the dorsal, ventral, medial and lateral cortical regions of 1-, 2- and 3-month-old rabbits. (b) Regional vascular canal density across the four cortical orientations.

Supplementary Figure 2 | Regional osteon morphology after AIMD. (a) Representative histological images from the dorsal, ventral, medial and lateral cortical regions of Control and AIMD rabbits. (b) Additional morphometric parameters of primary and secondary osteons, excluding osteon count.

Supplementary Figure 3 | Cortical bone micro-CT findings after AIMD. (a, b) Representative longitudinal sections, cross-sections and three-dimensional cortical bone reconstructions from Control and AIMD rabbits. (c–e) Quantification of cortical thickness, cortical area and tissue mineral density.
